# Comparison of the adjuvant effect of AS01, AS03 and alum on the immune response to GMP-grade H5N1 antigen in guinea pigs

**DOI:** 10.1038/s41598-026-65812-x

**Published:** 2026-08-20

**Authors:** Emmelie Eckhardt, Josephine Friedrich, Wiebke Lange, Julia Sehl-Ewert, Timm Harder, Jens Teifke, Ger van Zandbergen, Max Bastian

**Affiliations:** 1 Friedrich- Loeffler-Institut, Südufer 10, 17493 Greifswald, Insel Riems Germany; 2 Paul-Ehrlich-Institut, D-63225 Langen, Germany

**Keywords:** AS01, AS03, Adjuvants, Influenza, Guinea pig, H5N1, Biotechnology, Diseases, Drug discovery, Immunology, Microbiology

## Abstract

Adjuvants function as an enhancer and activator of the immune response and can be of pivotal importance for the efficacy of vaccines. AS01, which is a liposome formulation spiked with the saponin derivate QS-21 and the detoxified lipopolysaccharide MPL-A, and AS03, which is a squalene-based oil in water emulsion that contains antioxidant tocopherol, are modern, highly potent adjuvants that became integral to innovative vaccines. In the current study, guinea pigs –a well-established small animal model for human influenza-infections– were subcutaneously vaccinated with an influenza H5N1-antigen formulated with AS01, AS03, aluminium hydroxide or left unformulated. The induction of T- and B cells specific for the influenza antigen was assessed in dependence of the antigen formulation. H5N1-specific T cell responses were observed with all adjuvant formulations, but after six months were highest in the AS01- and AS03-immunized groups. The influenza specific antibody titres were highest in AS03-vaccinated guinea pigs. Importantly, AS03- and AS01-formulated influenza antigen also induced crossreactive antibodies inhibiting related H5N3- but not a distant H5N8-influenza strain. At the same time, it was observed that animals, which received the egg-produced H5N1 split antigen in combination with AS03, mounted xenoreactive, opsonizing antibodies that bound to chicken fibroblasts. No reactivity was observed to human fibroblasts. In summary, our results confirm the superior adjuvanticity of AS03 and of AS01 as compared to conventional alum or non-adjuvanted formulations. The presence of xenoreactive antibodies after AS03 vaccination was an unexpected observation. Further investigations will be required to appreciate the significance and identify the mechanisms of this findings.

**Supplementary Information:**

The online version contains supplementary material available at 10.1038/s41598-026-65812-x.

## Introduction

Adjuvants have a profound influence on vaccine induced immune responses. In particular, for inactivated or subunit vaccines the immune enhancing effect is often important to achieve a sufficient vaccine efficacy^1^. The way how adjuvants function is not entirely understood. Conventional adjuvants, such as aluminium salts, are believed to have a depot-effect and lead to enhanced uptake of particulate antigen by dendritic cells^2^. In addition, it is known that the aluminium salts can activate the inflammasome in antigen presenting cells and thereby further enhance the immune response^3^.

This dual mode of action, the antigen delivery and the immunostimulation, has also been described for modern adjuvants, such as the AS01- and AS03-formulation that have been developed by and are proprietary to GlaxoSmithKline (GSK)^4^. AS01 is a liposome-based vaccine adjuvant system containing two immunostimulants: 3-O-desacyl-4’-monophosphoryl lipid A (MPL) and the saponin QS-21. AS03 is an oil in water (o/w) emulsion that contains antioxidant tocopherol. Both adjuvants have been thoroughly tested in clinical trials and are part of licensed vaccines^5^. AS01 forms the basis of the successful malaria vaccine Mosquirix and the shingles vaccine, Shingrix. AS03 has been used in the H1N1-pandemic emergency vaccine Pandemrix^6^. It was also part of the H5N1-influenza vaccines Pumarix^7^ and Prepandrix^8^. Furthermore, it is a constituent of several Covid-19 vaccine candidates^9–12^. One of these candidates, VidPrevtyn Beta, most recently received a marketing authorization by the European Medicines Agency^13^. Seasonal influenza vaccines are often produced without adjuvant, but the amount of antigen that is required to elicit equivalent immune responses is several times higher compared to adjuvanted vaccines. The availability of embryonated specific pathogen free chicken eggs is an important bottleneck in influenza vaccine production^14^. During the H1N1-pandemic the antigen limitation made it necessary to produce adjuvanted vaccines. Pandemrix, the adjuvanted GSK vaccine conferred excellent protection and helped to confine the virus spread. It was estimated that approximately 30.5 million doses of Pandemrix were administered and the vaccine proved to be efficacious and to have an acceptable safety profile in various populations^15^. However, in some countries an increased risk of narcolepsy was detected. Narcolepsy is an autoimmune disease that is due to immune-induced hypocretin-deficiency in a hypothalamic structure, which is responsible for the sleep/wake rhythm^16^. No specific target antigen, which might have explained the putative autoimmune reaction, was unambiguously identified in the vaccine formulation. By contrast, Cohet and colleagues hypothesized in a recent paper that a cross-reactive epitope in the H1N1-antigen caused hypocretin-specific autoimmunity^15^. This is in line with reports of a higher incidence of narcolepsy during the H1N1 pandemic in China and Taiwan, where Pandemrix had not been used^17^.

In a comprehensive in vitro approach, we recently investigated the mode of action of individual adjuvant components on human immune cells^18^. In the current study, we extended these efforts to analyse the effect of complete adjuvant formulations on the priming and imprinting of adaptive immune responses. AS01 and AS03, the two adjuvant systems that are already part of licensed human vaccines, were head-to-head tested and compared to aluminum-hydroxide. Since during the swine flu pandemic a lot of clinical experience was collected with the AS03 adjuvanted H1N1-vaccine, we tested the adjuvants in combination with an influenza antigen. As small animal in vivo model we used guinea pigs, first of all because they are a well-established small animal model for human influenza^19–21^ and second because it is now generally accepted that experiments in inbred mouse strains need to be complemented with studies in other, outbred animal models^22,23^. So, aim of the current study was to compare the effect of conventional alum and the innovative adjuvant formulations AS01 and AS03 on (i) the antigen-specificity of vaccination induced T- and B-cell-responses and (ii) the quality of the induced immune responses. The study was conducted in a close collaboration between the Paul-Ehrlich-Institute, the German Federal Institute for Vaccines and Biomedicines, and the Friedrich-Loeffler-Institut, the Federal Research Institute for Animal Health.

## Materials and methods

### Animals and vaccination schedule

Thirty female SPF- Dunkin Hartley guinea pigs were obtained from Charles River Laboratories (Sulzfeld, Germany) at an age of 3 months. At the institute the animals were kept under conventional conditions in accordance with Appendix A of the European Convention for the Protection of Vertebrate Animals Used for Experimental and Other Scientific Purposes as adopted by the German Animal Welfare Legislation. The animals were housed in groups of two or three animals in open cages on dust-free wooden bedding. The animals had free access to dry pellets and water. Daily they were offered vegetables. The cages were enriched with a plastic refuge and hay for chewing and concealment. The facility is air-conditioned and has an average temperature of 20 °C. The light regimen follows a natural day and night cycle. After an acclimatization phase of two weeks 6 animals per group were subcutaneously vaccinated with 1.5 µg GMP-grade H5N1 antigen, as described previously^24^. All reagents were generously provided by GSK. The split antigen was produced in embryonated chicken eggs and originated from Influenza A virus strain, A/Indonesia/05/2005^7^. (The GSK pandemic Influenza A vaccines, Pumarix and Prepandrix, incorporated the same Influenza A antigen and were formulated with AS03. In the EU the vaccines were licensed in 2008 and 2011, respectively. In the meantime, both marketing authorisations have been withdrawn by the company. A third very similar AS03-adjuvanted, prepandemic H5N1-vaccine, Adjupanrix, is still authorised. It contains a closely related H5-antigen derived of strain A/Vietnam/1194/2004.) The antigen dose was either resuspended in 250 µl 0.9% sodium chloride solution or formulated in 250 µl with 0.2% aluminium-hydroxide, with 250 µl of GSK-adjuvant AS01 or AS03. The alum formulation was provided ready-to-use, AS01 and AS03 were mixed with the antigen immediately prior to use for 5 min at medium speed on an orbital shaking table according to the instructions. The control group received 250 µl sodium chloride. The doses were applied three times to the left chest area, approximately 3 cm left to the midline towards the axillary area with an interval of 21 days. Vaccines can be parenterally administered via the subcutaneous or the intramuscular route. Both approaches induce very similar immune responses and are sometimes used synonymously^25–29^. Since animal welfare guidelines preclude in guinea pigs to inject a volume of 250 µl intramuscularly, it was decided to deviate from the recommended route for the use in humans and apply the vaccine doses in the current study via the subcutaneous route. Every day, all animals were carefully observed by trained animal care takers. Once a week and every second day during the first week after the vaccination the animals were examined by a veterinarian. The examination included a score-based assessment of the general appearance and behaviour and the measurement of the body weight and rectal temperature. In addition, the injection sites were manually palpated. After completion of the animal experiment, the animals were euthanized under deep anaesthesia by carbon dioxide inhalation. The animal trial protocol was approved by the competent authority, the Landesamt für Landwirtschaft, Lebensmittelsicherheit und Fischerei, Mecklenburg-Vorpommern (7221.3-1-010/20).

### Analysis of T cell responses

Before vaccination and thereafter at day 21, 42, 63 and 148 blood samples were obtained from anesthetized animals by cardiocentesis, i.e. cardiac puncture. Guinea pigs were anesthetized using Ketamin (Livisto, Germany) and Xylazin (CP-Pharma, Germany). Mononuclear cells were isolated by Ficoll gradient centrifugation using Ficoll-Paque Plus (GE Healthcare). For the analysis of antigen specific T cell proliferation, the cells were stained with the green fluorescent dye, carboxy-fluorescein-succimidyl-ester (CFSE, Enzo Life Sciences) at a 5 µM working concentration. 1 × 10^5^ cells were seeded in 96 well round-bottom well plates and stimulated with 1 µg/ml H5N1 protein antigen or a peptide cocktail spanning a large part of the H5-antigen (see supplemental Table 1 for more details). Phytohemagglutinin (PHA, Oxoid Germany) served as a positive, Ovalbumin (OVA, Sigma-Aldrich) as a negative control. 5 days after stimulation, CFSE dilution was measured by flowcytometry as a measure for T cell proliferation. 63 days after vaccination cells were additionally investigated for antigen-specific cytokine expression. Cells were stimulated as described above with H5N1-antigen, OVA or left unstimulated. After 24 h the cells were harvested, RNA from stimulated and non-stimulated controls was isolated using Trizol reagent. Subsequently, the expression level of a selected panel of T cell cytokines was analysed by quantitative RT-PCR using the SYBR-green reagents (QIAGEN) according to manufacturer’s recommendations. The relative expression level was calculated for each gene of interest in relation to the ß-Actin housekeeping control, according to the following equation: Transcript level = 1000 × 2^(Ct(ß−Act)−Ct(gene of interest))^.

### Analysis of influenza-specific antibody responses

From all animals plasma samples were stored at each time point and analysed for influenza specific antibodies using three different approaches: (i) A commercial H5-specific competition ELISA was obtained from ID-VET. Plasma samples were tested according to manufacturer recommendations. (ii) ID-VET H5-precoated ELISA plates were used in a sandwich-ELISA-format to detect H5-specific guinea pig IgG. (iii) As a surrogate of neutralizing activity, the plasma was tested by hemagglutinin-inhibition (HI) test using virus antigen from an Indonesian H5N1-strain (A/ck/Indonesia/R60/2005 (HP H5N1); sequence accession no. in EpiFlu: EPI_ISL_2917) that was closely related to the 2005 virus used for the production of the prepandemic vaccine. Cross-reactivity in HI assays was evaluated with virus antigen produced and from a most recent H5N8-isolate of the goose/Guangdong lineage, clade 2.3.4.4b (A/barnacle goose/Germany-SH/2020AI02167/2020 (HP H5N8), sequence accession no. in EpiFlu: EPI_ISL_614400) and from a more distantly related low pathogenicity H5N3-strain of Eurasian lineage (A/Eurasian teal/England/7394 − 2805/06 (H5N3)). The first two antigens were produced by the National Reference Laboratory at the Friedrich-Loeffler-Institut, Isle of Riems, the latter by the European Refence Laboratory for Avian Influenza, Padova, Italy. The antigens were produced in the frame of this study from allantoic fluid of inoculated embryonated chicken eggs by inactivation with beta-propiolactone. The test was performed as previously described using four hemagglutinating units of antigen^30^.

### Analysis of xenoreactive antibody responses

Plasma samples were additionally used to test for vaccine induced-xenoreactive binding. The influenza split antigen had been produced in hen-eggs. Primary chicken embryo fibroblast cells and chicken fibroblast cell line, DF1, was therefore used to test for anti-chicken reactivity. Two immortalized human cell lines, the monocyte cell line THP-1 and the fibroblast cell line, HEK293, were used to assess anti-human reactivity. For the detection of cell-opsonizing antibodies, 1 × 10^5^ cells were incubated with 20 µl of the corresponding plasma and washed. Subsequently, cells were incubated with a FITC-conjugated anti-guinea pig IgG antibody (AbD-serotec). Cell-bound antibodies were then quantified by flowcytometry, using a MACSQuant Analyzer.

For individual experiments, DF1-cell-bound antibodies were detached from the cells using an acidic buffer, as described elsewhere^31^. In brief, 1 × 10^7^ DF1 cells were incubated with the corresponding plasma. Unbound antibodies were removed by extensive washing. Cell-bound antibodies were then detached using 0.12 M Citrate buffer (pH 2.5; Sigma-Aldrich). The cells were removed by centrifugation and the antibody containing supernatant was immediately neutralized with 1 M Tris-HCl buffer (pH 9.0; Sigma-Aldrich). Subsequently, the detached antibodies were analysed for H5-reactivity by ELISA or by flowcytometry for binding to the DF1 cell line, as described above.

Immunoprecipitation was employed to visualize the target antigen of xeno-reactive antibodies, as described elsewhere^32^. Briefly, 6 × 10^6^ DF1 cells were extracellularly biotinylated using 2 mM Sulfo-NHS-LC-Biotin (Thermo Scientific) and incubated with 300 µl plasma from control or H5N1/AS03 vaccinated animals. After extensive washing the cells were lysed in Lysisbuffer (150 mM NaCl, 50 mM Tris, 5mM EDTA) with or without 0.1% Digitonin (Sigma-Aldrich) using a Branson-Sonifier. Antibody-bound antigen was immunoprecipitated using 100 µl ProtG-Sepharose (GE Healthcare). After intensive washing in the respective lysis buffer, the precipitate was resuspended in 25 µl Laemmli-buffer and loaded on 15% poly-acrylamide gels. After SDS-PAGE the proteins were transferred to nitrocellulose membranes. Cell-surface proteins were revealed by Streptavidin-HRP conjugate (Dianova) using AEC chromogen (Sigma-Aldrich).

### Statistics

GraphPad Prism version 8.1.0 was used to analyse and visualize the data. Quantitative data are expressed as group means. Error bars indicate the standard error of the mean or standard deviation, as indicated. Data were tested for normal distribution using Shapiro-Wilk and Kolmogorov-Smirnov test. Results from proliferation-assays were analysed by single sample T Test or Fisher’s LSD test, as indicated in the figures legend. P-values are expressed as follows: *, *p* < 0.05; **, *p* < 0.01, ***, *p* < 0.001 and ****, *p* < 0.0001. Data without symbol did not reach significance.

## Results

### Application and tolerability

**Fig. 1:**
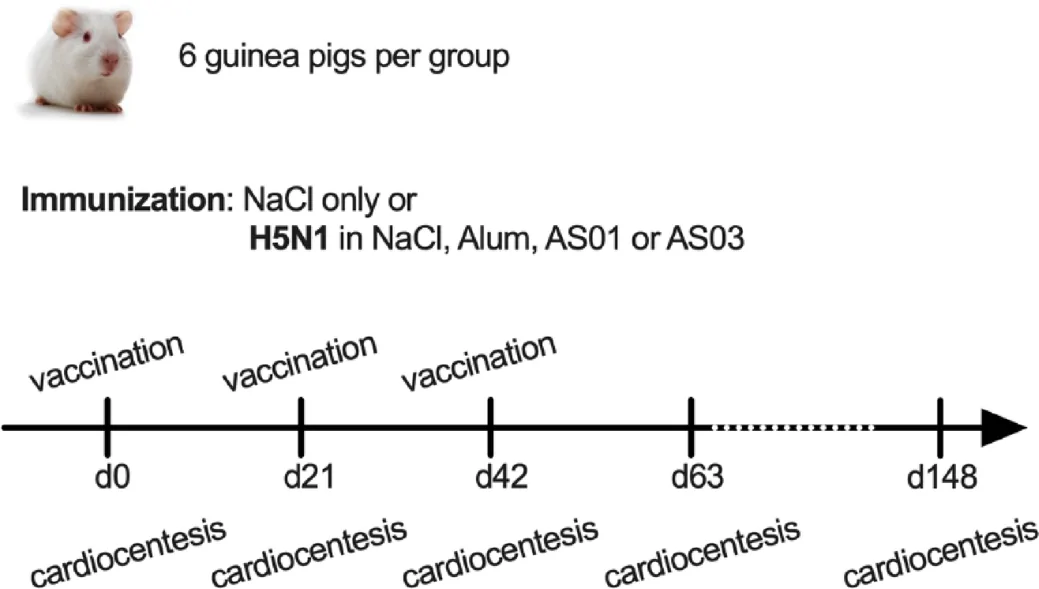
Schematic representation of the animal trial design. Six guinea pigs per group were three times vaccinated with H5 antigen in saline, adsorbed to alum or formulated with AS01 or AS03. The control group received only saline. At various time points blood was obtained by non-terminal cardiocentesis.

By subcutaneous route, guinea pigs received three consecutive vaccinations with GMP-grade H5N1-antigen that was either solubilized in physiological sodium chloride solution or adsorbed to aluminium-hydroxide or was formulated in one of the innovative GSK-adjuvants AS01 or AS03 (see Fig. 1). The control group was administered only sodium chloride solution. Throughout the study, the animals were carefully observed daily by trained animal caretakers. During the first week after vaccination, every second day the animals were examined by a veterinarian. Body weight and temperature was taken, and the site of injection was palpated. Half an hour after the first vaccination one animal of the AS01 group died due to pericardial tamponade, a direct consequence of the cardiocentesis, as confirmed by necropsy. Apart from this single fatality all manipulations and vaccinations were well tolerated. The animals showed normal behaviour and appearance throughout the study. There was no change in the body temperature after vaccination, and there was no difference in the weight gain between the five groups. However, after the second and more so after the third vaccination the two groups that received AS01- and in particular the AS03-formulated antigens developed swellings at the injection site. The skin and fur remained intact, no pain indicative reaction from the guinea pigs could be observed during palpation and after another two weeks the swellings shrank in size. 148 days after the first and 106 days after the last vaccination the animals were euthanized and dissected.

### T cell responses to the vaccination antigens

**Fig. 2:**
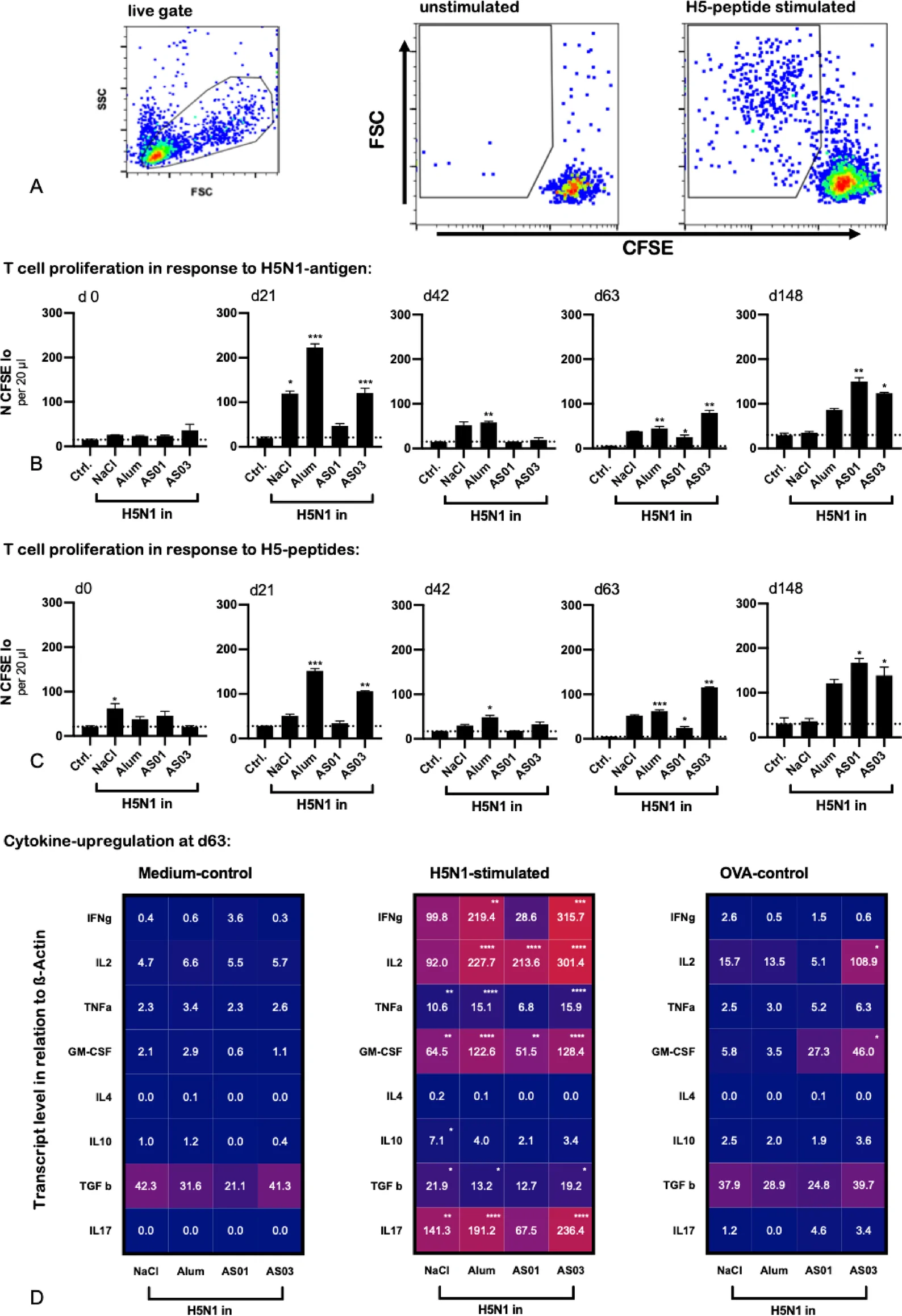
Adjuvanted H5N1-formulations elicit sustained T cell responses: At the indicated timepoints blood was obtained from vaccinated animals. Blood mononuclear cells were isolated by Ficoll gradient centrifugation. Antigen-specific cell proliferation was tested by CFSE dilution assay. (A) As an example, unstimulated or stimulated cells from a H5-AS03 vaccinated guinea pig are shown after five days of incubation. The H5N1 split antigen (B) or a peptide pool spanning the conserved C-terminal part of the H5-antigen (C) was used to stimulate the cells. Results are shown as the absolute number of CFSE low, proliferated cells in 20 µl cell culture medium. Black bars represent the group mean for the indicated vaccination group. Error bars represent the standard error of the mean. The dotted line represents the level of proliferation observed in animals having received only NaCl. Asterisks indicate the level of significance as calculated by single sample T Test in relation to the control. (D) 63 days after the first vaccination blood mononuclear cells were stimulated with H5N1-split antigen or OVA. After 24 h RNA was isolated and transcription levels of selected T cell cytokines were determined by quantitative RT-PCR. Transcription levels were calculated in relation to the ß-Actin expression level. Columns of the heatmaps represent the four vaccination groups, the rows stand for the respective gene. Figures displayed in the fields indicate the group mean over six animals of the respective transcript level. The left heatmap shows the transcription level in the absence of antigen-stimulation, the middle shows transcription levels in response to H5N1-split antigen and the right panel shows the response after OVA-stimulation. Asterisks indicate the level of significance as calculated by Fisher’s LSD test in comparison to the unstimulated control.

The antigen-specific proliferation to vaccine antigens was assessed by a CFSE-based proliferation assay. Cells were stimulated either with GMP-grade H5N1-split-antigen that had been used for the immunization or with a 15-mer peptide pool spanning the H5-antigen. After five days CFSE-dilution was measured by flowcytometry to determine cell proliferation. The gating strategy is depicted in Fig. 2A. 21 days after the first vaccination all animals that had received H5N1 antigen responded with cell proliferation after split protein or peptide stimulation. In the case of split protein significance was reached for the group that had received the H5N1 antigen in saline, adsorbed to alum or formulated with AS03. For the group that had received the antigen in the AS01 formulation the group mean was not significantly different from the control. A similar pattern was observed with peptide stimulation. However, in this case the group that received the antigen in saline did not reach significance, either. During the course of the experiment the proliferative response showed some contraction, because after 42 and 63 days the number of proliferated cells was clearly lower compared to the time point after 21 days. For the group that had received the antigen formulated in AS03 the T cell proliferation recovered after 63 and even more so after 148 days. It is of note that at this time point the proliferative response of animals that had received the antigen in AS01 exceeded the response of the other groups (Fig. 2B and C). To investigate the cytokine repertoire expressed by vaccine induced T cell responses, we measured the upregulation of a small but comprehensive panel of T cell genes 63 days after the first vaccination. To this end cells were stimulated with H5N1 split antigen or OVA as a control. After 24 h RNA was harvested and the transcription level of the specific cytokines was measured by quantitative RT-PCR. The results are depicted in Fig. 2C: Each column in the heatmaps represents one of the four groups that had received H5N1 antigen. Rows represent the corresponding genes. The figures in the individual cells represent the group mean of the specific transcription level, as calculated in relation to the ß-Actin housekeeping gene. A dark blue colour indicates low level, red colour indicates high levels of expression. The left heatmaps in Fig. 2C shows the results for the unstimulated, the middle for the H5N1- and the right heatmap for the OVA-stimulated cells. In the absence of an antigen stimulus a low relative expression level was observed for all cytokines. Only for TGF-ß there was an intermediate transcription level. Upon H5N1-stimulation a clear upregulation of prototypic Th1-cytokines, such as IFN-g, IL2 and GM-CSF, and of the Th17-cytokine IL17 was detected. At the same time there was a reduction of the relative expression level of TGF-ß. It is noteworthy that for all groups a similar cytokine pattern was revealed. There were no obvious differences, although quantitatively the level of cytokine upregulation paralleled the level of T cell proliferation after 63 days. The highest expression of IFN-g and IL17 was observed in animals that received the AS03-formulated antigen. The lowest was seen in animals that received the AS01-formulation. The transcription level after OVA-stimulation was very comparable to the non-stimulated control. However, it is of note that in animals that received the AS03-formulation a marked upregulation of IL2 and GM-CSF was observed. With AS01-vaccinated animals at least some GM-CSF-upregulation in response to OVA was detected (Fig. 2D).

### Influenza specific antibodies

**Fig. 3:**
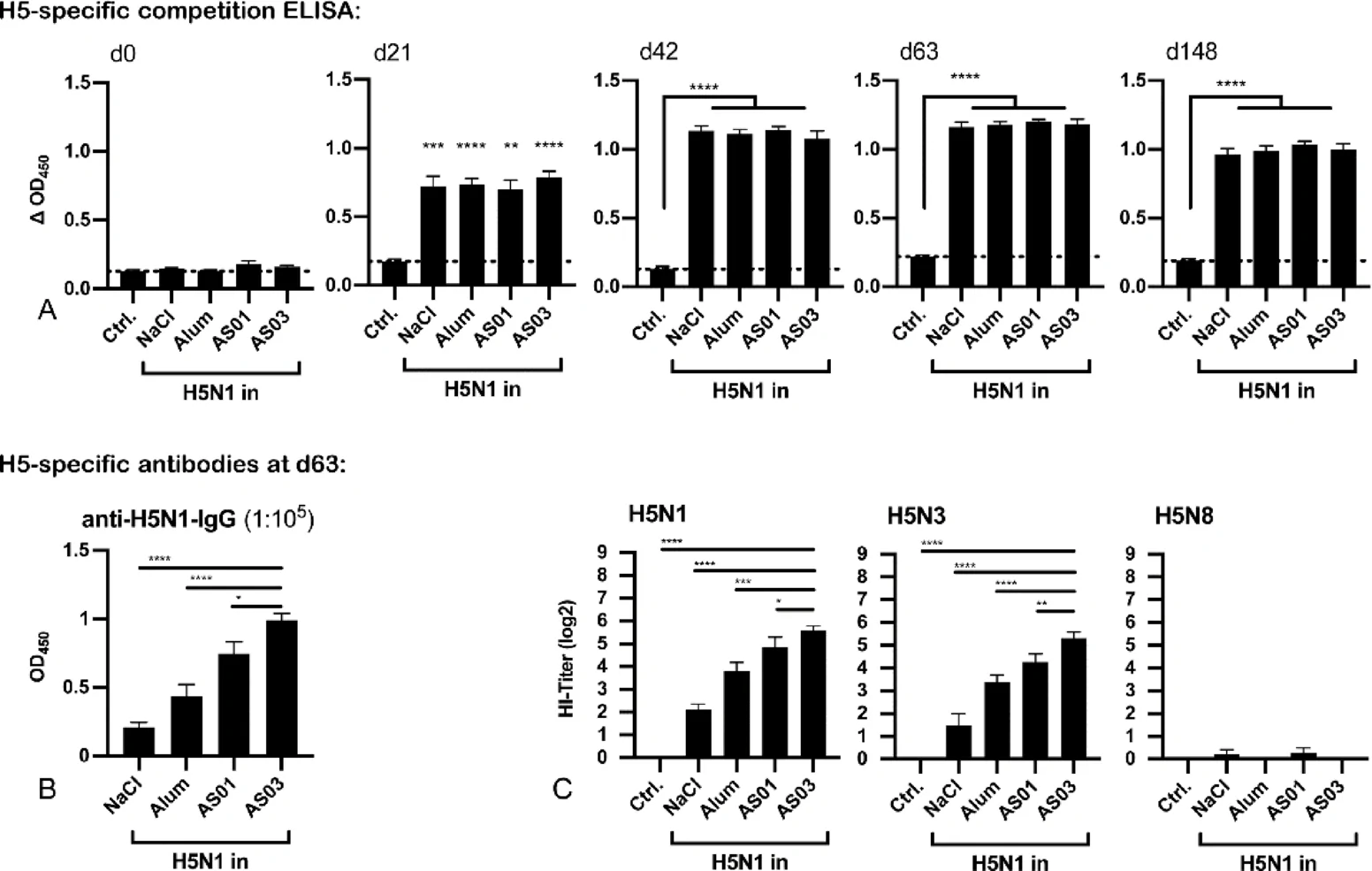
Adjuvanted H5N1 elicits potent H5-neutralizing antibodies: (**A**) At the indicated timepoints blood was obtained from vaccinated and control animals. H5-specific antibody levels in the respective plasma were determined by competitive ELISA. Results were calculated as ∆OD_450nm_ by subtracting the test from the negative control. Black bars represent the group mean, error bars the standard error of the mean. The dotted line indicates the control signal. Asterisks indicate the level of significance as calculated by single sample T test in relation to the control. (**B**) Conventional sandwich ELISA was used to analyze serial dilutions of plasma from H5N1 vaccinated animals. Results are shown as OD_450_ for the highest dilution (1:1 × 10^5^). (**C**) Haemagglutinin inhibition test (HI) was used to test for the neutralizing activity of immune plasma. HI-Titres represent the log 2 of the reciprocal level of the highest dilution showing inhibition. The left panel shows inhibition against a virus strain closely related to the production strain (H5N1). The graph in the middle shows the inhibition against a more distantly related virus (H5N3). The right graph shows the results for a most recent virus isolate (H5N8). Black bars represent the group mean, error bars the standard error of the mean. Asterisks indicate the level of significance as calculated by Fisher’s LSD test in comparison to the AS03-vaccinated animals.

A commercial competition ELISA kit was used to detect H5-specific antibodies. Plasma from all time points and from all animals was tested. After 21 days all vaccinated groups showed a significant reduction in the signal of the competitive ELISA. The inhibitory effect of H5-specific antibodies increased after the second dose, reached a plateau after 63 days and began to wane after 148 days (Fig. 3A). The competitive ELISA is designed to qualitatively show the presence of H5-specific antibodies. To reveal quantitative differences between vaccination groups, the plasma obtained after 63 days were additionally investigated in a sandwich ELISA using the same H5-precoated ELISA plates. Serial dilutions of all plasma were tested. The 1:10^5^ dilution revealed that animals that had received the antigen in AS03 had significantly higher antibody levels than the other groups. Although only low T cell responses had been observed with AS01 vaccinated animals, they showed the 2nd -highest level of H5-specific antibodies (Fig. 3B). Titration of hemagglutination-inhibiting antibodies in 63-day plasma samples by HI-test confirmed the sandwich-ELISA results: First, the plasma was tested against a H5N1-antigen that was derived from a virus strain very closely related to the virus used for vaccine production (“homologous” antigen). Plasma from animals vaccinated with the AS03-formulation showed a significantly higher HI titre compared to all other groups. The same was observed when the plasma was tested against heterologous virus antigens from a more distantly related H5N3-virus. No inhibition whatsoever was observed, when the plasma was tested against antigen from a most recent H5N8-virus isolate (Fig. 3C).

### Xenoreactive antibodies

**Fig. 4:**
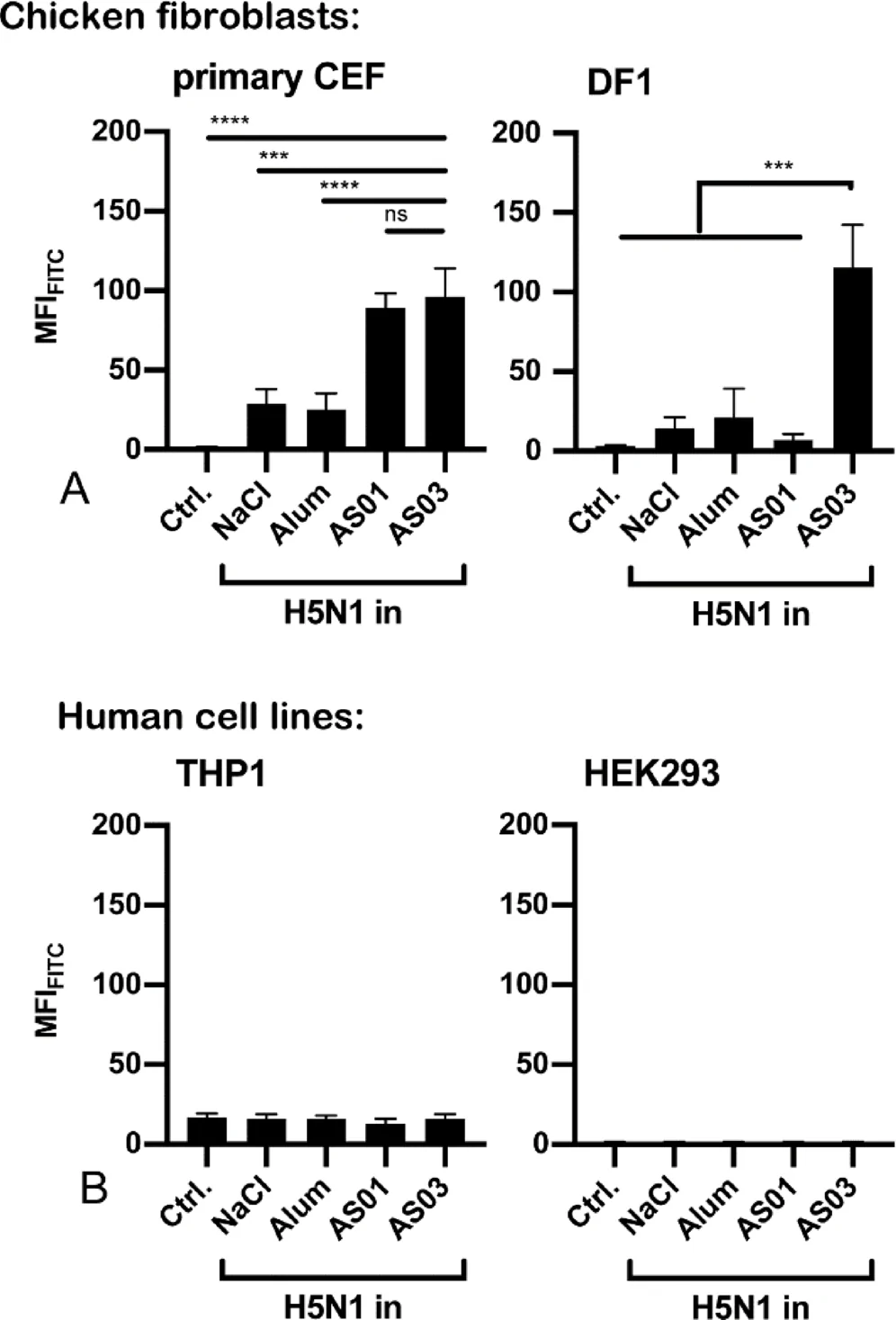
Adjuvanted H5N1 elicits xenoreactive antibodies: (**A**) After 63 days plasma was obtained from vaccinated and control animals and tested by flow cytometry for antibodies binding to the cell surface of primary chicken embryo fibroblasts (CEF, left panel) or a permanent chicken fibroblast cell line (DF1, right panel). (**B**) The same plasma was tested against a permanent human monocyte (THP1, left panel) or fibroblast cell line (HEK293, right panel). Results indicate the mean fluorescence intensity (MFI) as measured by flowcytometer. Black bars represent the group mean, error bars the standard error of the mean. Asterisks indicate the level of significance as calculated by Fisher’s LSD test in comparison to the AS03-vaccinated animals.

In a final step, it was tested, whether the guinea pigs mount an immune response towards production derived cellular antigens. Of particular relevance are opsonizing antibodies that bind to the cell surface proteins of cells used to produce the antigens. To assess their opsonizing activity plasma obtained 63 days after the first vaccination was tested in a flowcytometric approach. Primary chicken embryonic fibroblasts (CEF) or cells of a permanent chicken fibroblast cell line (DF1) were incubated with the respective plasma. Cell-bound guinea pig IgG was revealed by FITC-conjugated secondary antibodies. All groups that had received the H5N1-antigen showed antibodies against primary CEFs. However, the two groups that had been vaccinated with one of the innovative GSK-adjuvants AS01 or AS03 showed significantly higher binding (Fig. 4A, left panel). A more defined and reproducible read-out allowed the permanent DF1 cell line: Here, only the group that had received the AS03-formulation displayed binding (Fig. 4A, right panel). No reactivity whatsoever was observed to one of the two human cell lines tested (Fig. 4B).

**Fig. 5:**
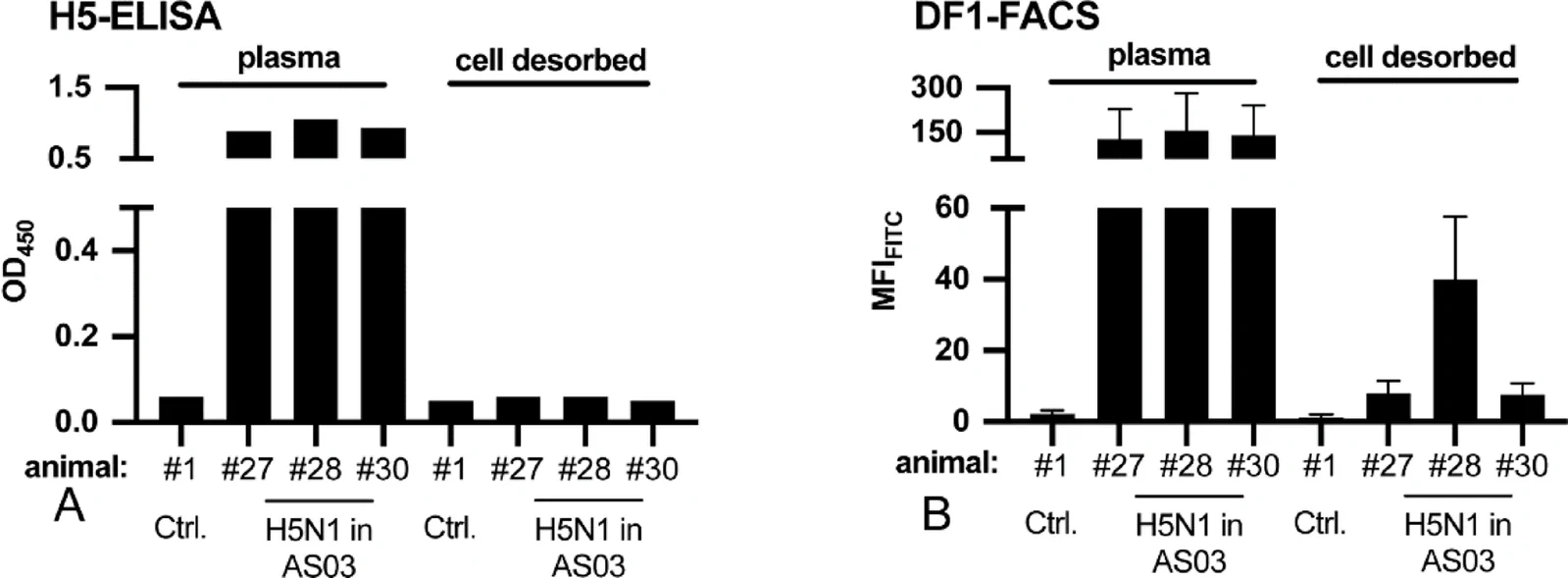
Cell reactive antibodies are not H5-related: Cell reactive antibodies were obtained by incubating DF1 cells with plasma from three AS03-vaccinated and one control animal. After extensive washing cell bound antibodies were desorbed from the cell surface and then tested for their antigen specificity: (**A**) Plasma and cell desorbed antibodies were tested for H5-binding. The first four bars indicate the level of binding for the plasma, the right side of the graphs shows H5-binding for the corresponding cell desorbed antibodies. Black bars represent the OD_450_-level as measured by conventional sandwich ELISA. (**B**) Subsequently binding to the DF1 cell was tested by flow cytometry. Again for antibodies binding to the cell surface of primary chicken embryo fibroblasts (CEF, left panel) or a permanent chicken fibroblast cell line (DF1, right panel). Results indicate the mean fluorescence intensity (MFI) as measured by flowcytometer. Black bars represent the mean for duplicate measurements, error bars the standard deviation.

To prove that the cell binding antibodies were not cross-reactive to the H5-antigen an affinity purification was performed: Antibodies were bound to the DF1-cell line. After extensive washing, the antibodies were eluted using an acidic buffer. The cell desorbed antibodies were subsequently tested for H5- or DF1-specificity. Figure 5 shows the results: In panel A the H5-binding is assessed for plasma from one control animal and three animals that received the AS03-formulated H5N1 antigen. High levels of H5-binding antibodies are found in the plasma of the three vaccinated animals. After affinity purification no H5-binding whatsoever is left (Fig. 5A). The same set of plasma and cell desorbed antibodies was tested for binding to the DF1 cell line: Pronounced binding was observed both, with the plasma samples and with the cell desorbed antibodies. Although the binding with the cell desorbed antibodies was clearly lower -probably due to a dilution effect- it was still significant when tested by One sample Wilcoxon test (Fig. 5B).

**Fig. 6:**
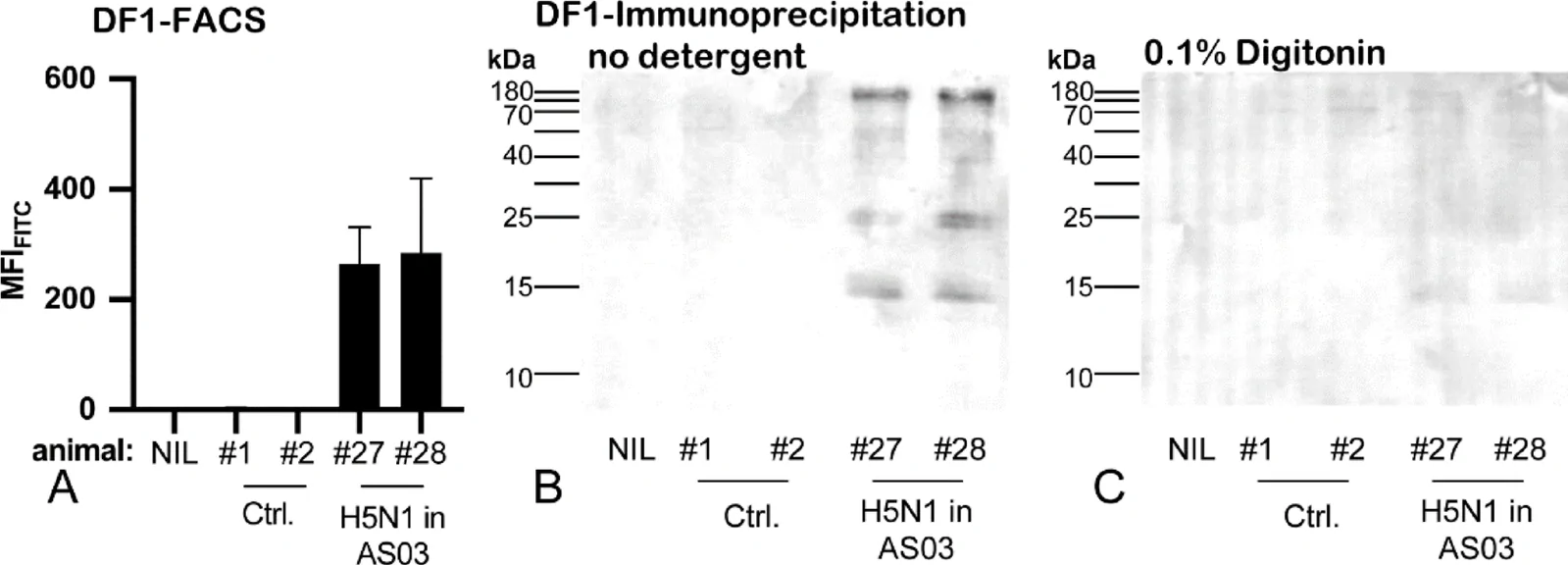
Cell reactive antibodies recognize a 15 kDa antigen: (**A**) A panel of plasma from two control and two AS03-vaccinated animals was tested by flow cytometry for binding to the DF1 cell. Results indicate the mean fluorescence intensity (MFI) as measured by flowcytometer. Black bars represent the mean for duplicate measurements, error bars the standard deviation. The same plasma panel was used to immunoprecipitate extracellularly biotinylated surface molecules of the DF1 cell line lysed in the absence (**B**) or presence (**C**) of 0.1% Digitonin lysis. After 15% SDS-PAGE proteins were transferred to a nitrocellulose membrane. Transferred, biotinylated proteins were revealed by Streptavidin-HRP-conjugate using the AEC chromogen.

Immunoprecipitation was employed to further establish the cell surface binding. To this end two control plasma and two highly reactive plasma from AS03 vaccinated animals were selected. DF1 binding for the corresponding plasma is shown in Fig. 6A. DF1 cells were incubated with the respective plasma, extensively washed and lysed in the absence or presence of the mild detergent Digitonin. Antibody bound, biotinylated cell surface proteins were precipitated and analysed by western blotting. After incubation with plasma from both AS03-vaccinated animals a faint band at 15 kDa was revealed (Fig. 6B, C).

## Discussion

In the current study, we used outbred guinea pigs to investigate the in vivo effect of modern adjuvant systems. In this robust model that provides additional, more real-world insights into vaccination-induced immune responses than inbred mouse strains, we could confirm the superiority of AS03-induced anti-influenza responses. This contributes to our understanding of the role of adjuvants, which has ever been a research focus for vaccinologists trying to unravel “immunologist’s dirty little secrets”^33^. Although there is longstanding experience with potent adjuvants in the field of veterinary vaccinology^34^, for many decades the only adjuvants for human use were aluminium salts. Thus, it was an important milestone, when companies like GSK developed and incorporated modern, innovative adjuvants into their portfolio of licensed vaccines. The licensing of these adjuvants spurred comparative analyses and a lot has been learned about immune enhancement due to the modern formulations^35^. The AS03 formulation in pandemic influenza vaccines drew great attention to this new development. A comprehensive meta-analysis of preclinical, clinical and post-licensure data concluded that the AS03-formulated Influenza vaccines displayed an acceptable safety profile^15^. This was confirmed in independent clinical studies: AS03-formulated influenza antigens are associated with a higher incidence of local reactions in comparison to non-adjuvanted formulations, but these were observed to be short-lived and self-resolving. At the same time, several studies observed higher and broader-reactive antibody titres with the new adjuvant formulation despite lower amounts of antigen per dose^36,37^. These antibodies were found to have a neutralising effect across different clades and were long-lasting^38^. In particular during pandemic situations, when the availability of antigen is limited and emergency vaccination campaigns require accelerated vaccine production, the option to achieve superior immune responses with a reduced amount of antigen can be of utmost importance^14,39,40^. Accordingly, Sanofi Pasteur is about to exploit the virtues of the AS03 adjuvant effect in a most recently licensed protein based vaccine against SARS-CoV-2^13^. The superiority of the AS03-induced anti-influenza immune response, as observed in the current study, is well in accordance with observations made in other preclinical models^41^: Antibody titres are higher and last longer compared to non-adjuvanted or (to a lesser extent) alum-adjuvanted formulations (see also supplemental Fig S1). In addition, we could show that AS03-induced influenza antibodies show a higher degree of cross-reactivity across different haemagglutinin-subclades. With respect to cellular responses, robust antigen-specific T cell proliferation and T cell cytokine upregulation was observed. Both adjuvant systems induced prolonged and more sustained T cell memory compared to the non- or alum-adjuvanted controls. Interestingly, we noted an apparent depression of T cell responses every 21 days after booster immunization (i.e. at day 42 and day 63). By contrast at day 148, in all groups that received the antigen in an adjuvanted form, the level of T cell proliferation exceeded the preceding responses. One possible explanation could be that, after booster vaccination, T cells tend to get sequestered at the site of injection and are only able to reenter circulation, after the local inflammation and the antigen deposits have been resolved. A related phenomenon was probably observed when mice were vaccinated with an experimental anti-tumor vaccine^42^. Although in inbred mice alum is generally seen as an adjuvant inducing predominantly Th2-responses^43,44^, we observed a strong upregulation of Th1- and Th17-cytokines with only little quantitative differences between Alum- and AS03-vaccinated animals. Interesting was the low level of T cell proliferation during the first time points after immunization with AS01-formulated H5N1-split-antigen. However, at day 148, T cells of AS01-vaccinated guinea pigs showed higher levels of proliferation compared to the other groups. That the T cells were primed during the initial course of vaccinations is reflected by the fact that cells from AS01-vaccinated animals showed a highly significant upregulation of IL2 upon H5-stimulation, albeit not of INF-γ (see Fig. 2D). Why the response pattern was so different compared to alum- or AS03-induced T cells remains to be clarified.

In addition to the antigen-specific reaction to influenza antigens, a second focus of the current study was to investigate vaccination-induced side reactivities: Egg-produced split antigens contain varying amounts of egg-derived antigens^45–49^. Usually, these minimal, residual amounts are well tolerated. For example, Forsdahl and colleagues observed no adverse reactions after influenza vaccination even in children with severe egg-allergy^50^. Accordingly, in the case of AS03-formulated influenza vaccines there were no confirmed safety signals regarding an increased risk of anaphylaxis compared to other vaccines^15^. However, some years ago, we investigated cases of a bovine neonatal alloimmune syndrome, *Bovine Neonatal Pancytopenia* (BNP). Only calves from cows vaccinated with one strongly-adjuvanted, bovine pestivirus-vaccine developed the disease^51^. It was later shown that the causative maternal, vaccination-induced antibodies were the result of high levels of bioprocess impurities in the vaccine in combination with a very potent, veterinary adjuvant^31^. In light of these previous studies, we tested the possibility that minimal amounts of bioprocess impurities, which are not recognized in an adjuvant-free vaccine formulation, may become immunogenic when combined with a highly potent adjuvant. In the current study, we tested ovalbumin as a potential bioprocess related T cell antigen. We did not observe any proliferative response, but when we investigated the upregulation of T cell cytokine genes, only in cells from AS03-vaccinated animals, we found significantly elevated transcription levels of IL2 and GM-CSF in response to OVA. In line with the reference^15^, the two cytokines are not associated with type 1 hypersensitivity reactions, but the response indicates that the bioprocess derived antigen is present in the split antigen and is recognized in the context of the adjuvant. Similarly, when we investigated immune sera, we discovered antibodies that bound to cell surface antigens on primary chicken fibroblasts, both after vaccination with AS01- and AS03-formulated H5N1-antigen. With sera from AS03-vaccinated animals we observed antibody-opsonization of the permanent chicken fibroblast cell line, DF1. This allowed us to investigate the reactivity in more detail: In a criss-cross experiment using cell-desorbed antibodies, we could demonstrate that the anti-fibroblast reactivity was unrelated to viral antigens. We were unable to identify the cognate antigen, but by immunoprecipitation we could confirm that the recognized surface antigen has an apparent size of about 15 kDa. When the H5N1 split antigen was tested by westernblot using the same immune plasma samples from AS03 vaccinated guinea pigs, the main reactivity presumably corresponds to different multimeric forms of influenza hemagglutinin. This shows the high purity level of the split antigen. Whether the faint band close to the running front is related to the reactivity as detected by DF1 immunoprecipitation remains to be investigated (see Fig S2). Ongoing efforts are aiming at identifying the mechanism that could explain, how the adjuvant induces cell opsonizing antibodies. In any case, it is important to emphasize that, in the current study, there was no antibody-reactivity against one of the two human cell lines tested.

## Conclusion

In summary, we have confirmed the superiority of anti-influenza immune responses induced by modern adjuvant formulations, namely AS03, compared to conventionally adjuvanted preparations. This was the first demonstration in guinea pigs and is well in accordance with a wealth of published studies. At the same time, we demonstrate that the immune enhancing effect of potent adjuvants also affects the recognition of bioprocess related antigens. In view of the numerous studies reporting that AS03-formulated influenza vaccines are well tolerated, it is obvious that the clinical relevance of these unwanted reactivities is very limited. However, the presence of xenoreactive antibodies after AS03 vaccination was an unexpected observation. Further investigations will be required to appreciate the significance and identify the mechanisms of this findings.

## Supplementary Information

Below is the link to the electronic supplementary material.

Supplementary Material 1

Supplementary Material 2

Supplementary Material 3

Supplementary Material 4

Supplementary Material 5

Supplementary Material 6

## Data Availability

All data supporting the findings of this study are available within the article. Raw data are available from the corresponding author, upon reasonable request.

## Abbreviations

GSK: GlaxoSmithKline
GMP: Good manufacturing practice
CFSE: Carboxy-fluorescein-succimidyl-ester
PHA: Phytohemagglutinin
OVA: Ovalbumin
HI: Hemagglutinin-inhibition
CEF: Chicken embryonic fibroblasts
