# Supplementary material for: Comparison of the adjuvant effect of AS01, AS03 and alum on the immune response to GMP-grade H5N1 antigen in guinea pigs
Source: Sci Rep. 2026 Aug 20;16:26175. doi: 10.1038/s41598-026-65812-x (PMC13493994; doi:10.1038/s41598-026-65812-x)
Supplement: Supplementary file 1 [file 41598_2026_65812_MOESM3_ESM.pdf]

Fig S1 AS03-adjuvanted H5N1 elicits longer lasting H5-specific antibodies:

H5-specific sandwich ELISA (1:10⁴ dilution):

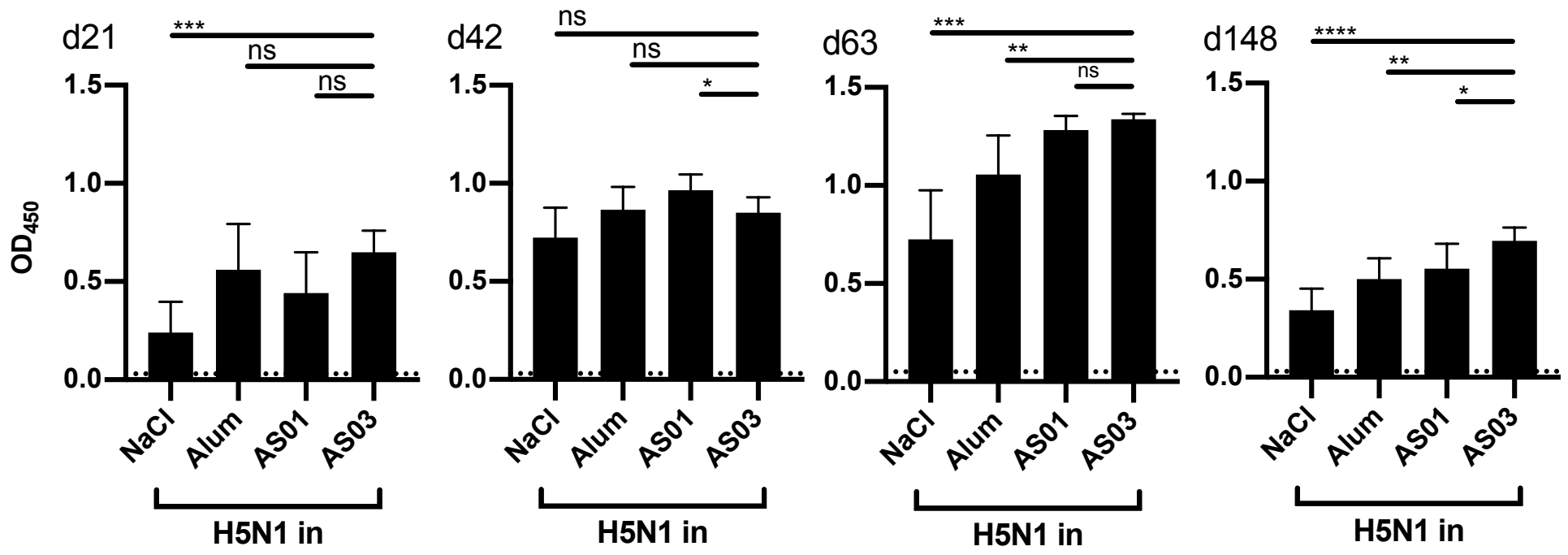

At the indicated timepoints, blood was obtained from vaccinated and control animals. H5-specific antibody levels in the respective plasma were determined by conventional H5-specific sandwich ELISA using serial dilutions of plasma from H5N1 vaccinated animals. Results are shown as OD_{450} for the 1:10^4 dilution. The dotted line depicts background level. Black bars represent the group mean, error bars the standard deviation. Asterisks indicate the level of significance as calculated by unpaired t test in comparison to the AS03-vaccinated animals.
