# Supplementary material for: Comparison of the adjuvant effect of AS01, AS03 and alum on the immune response to GMP-grade H5N1 antigen in guinea pigs
Source: Sci Rep. 2026 Aug 20;16:26175. doi: 10.1038/s41598-026-65812-x (PMC13493994; doi:10.1038/s41598-026-65812-x)
Supplement: Supplementary file 2 [file 41598_2026_65812_MOESM4_ESM.pdf]

```
(A/Indonesia/5/2005)     MEKIVLLLAIVSLVKSDQICIGYHANNSTEQVDTIMEKNVTVTHAQDILEKTHNGKLCDL     60
(A/Vietnam/1194/2004)    -------F----------------------------------------------------     60

(A/Indonesia/5/2005)     DGVKPLILRDCSVAGWLLGNPMCDEFINVPEWSYIVEKANPTNDLCYPGSFNDYEELKHL     120
(A/Vietnam/1194/2004)    -----------------------------------------V-------D----------     120

(A/Indonesia/5/2005)     LSRINHFEKIQIIPKSSWSDHEASSGVSSACPYLGSPSFFRNVVWLIKKNSTYPTIKKSY     180
(A/Vietnam/1194/2004)    -------------------S----L--------Q-KS--------------------R--     180

(A/Indonesia/5/2005)     NNTNQEDLLVLWGIHHPNDAAEQTRLYQNPTTYISIGTSTLNQRLVPKIATRSKVNGQSG     240
(A/Vietnam/1194/2004)    ------------------------K----------V-----------R------------     240

(A/Indonesia/5/2005)     RMEFFWTILKPNDAINFESNGNFIAPEYAYKIVKKGDSAIMKSELEYGNCNTKCQTPMGA     300
(A/Vietnam/1194/2004)    --------------------------------------T---------------------     300

(A/Indonesia/5/2005)     INSSMPFHNIHPLTIGECPKYVKSNRLVLATGLRNSPQRESRRKKRGLFGAIAGFIEGGW     360
(A/Vietnam/1194/2004)    ----------------------------------------R-------------------     360

(A/Indonesia/5/2005)     QGMVDGWYGYHHSNEQGSGYAADKESTQKAIDGVTNKVNSIIDKMNTQFEAVGREFNNLE     420
(A/Vietnam/1194/2004)    ------------------------------------------------------------     420

(A/Indonesia/5/2005)     RRIENLNKKMEDGFLDVWTYNAELLVLMENERTLDFHDSNVKNLYDKVRLQLRDNAKELG     480
(A/Vietnam/1194/2004)    ------------------------------------------------------------     480

(A/Indonesia/5/2005)     NGCFEFYHKCDNECMESIRNGTYNYPQYSEEARLKREEISGVKLESIGTYQILSIYSTVA     540
(A/Vietnam/1194/2004)    -----------------V-----D------------------------I-----------     540

(A/Indonesia/5/2005)     SSLALAIMMAGLSLWMCSNGSLQCRICI                                     568
(A/Vietnam/1194/2004)    --------V-------------------                                     568
```

Supplemental Table 1:

The table shows the amino acid sequence of the H5-hemagglutinin of Influenza A strain A/Indonesia/5/2005, corresponding to the antigen used in the current study, aligned to the H5-sequence of the closely related strain A/Vietnam/1194/2004 is aligned. A peptide pool consisting of 87 15mer peptides with an overlap of 11 amino acids spanning the conserved C-terminal part (dark red) was used to test for H5-specific T cell proliferation. Schematically, the peptides are depicted as blue lines.
