# Supplementary material for: Comparison of the adjuvant effect of AS01, AS03 and alum on the immune response to GMP-grade H5N1 antigen in guinea pigs
Source: Sci Rep. 2026 Aug 20;16:26175. doi: 10.1038/s41598-026-65812-x (PMC13493994; doi:10.1038/s41598-026-65812-x)
Supplement: Supplementary file 3 [file 41598_2026_65812_MOESM5_ESM.pdf]

Fig S2 Westernblotting reveals that the main component in the H5N1 split antigen corresponds to influenza A hemagglutinin:

H5N1 split antigen

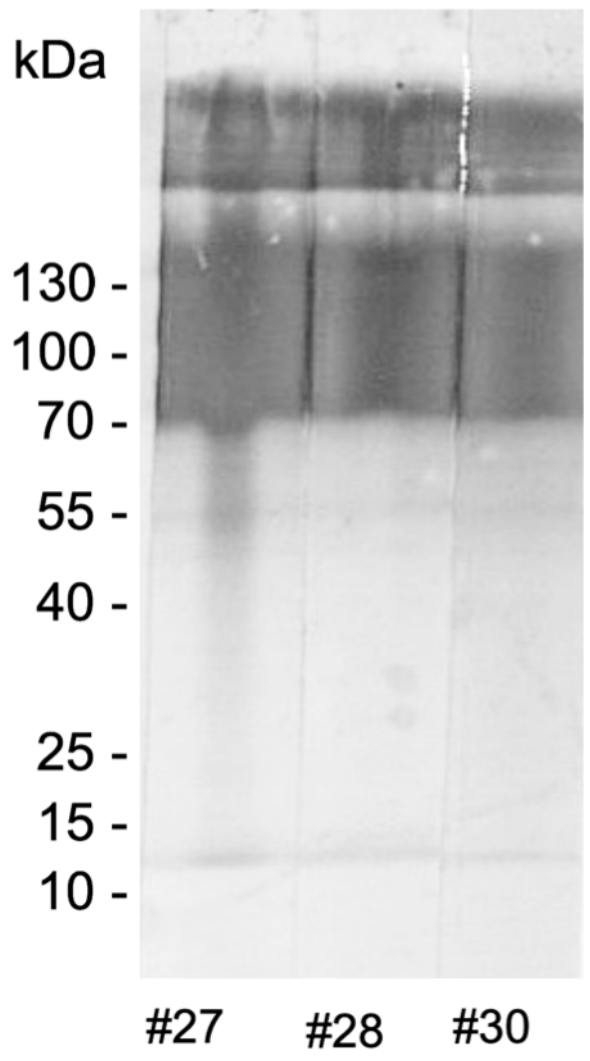

Plasma from three AS03-vaccinated animals, which were obtained at day 63 and showed a strong xenoreactive binding to the DF1 cell line, were tested by westernblot: 4 µg of the H5N1 split antigen were separated by non-denaturing SDS-PAGE and transferred to a nitrocellulose membrane. The membrane was cut into stripes. Stripes were individually incubated with 1:10 dilutions of the respective plasma. Antibody binding was revealed using an HRP-conjugated anti-gp-IgG secondary antibody.

In the split antigen, the main reactivity was found between 70 and 130 kDa. This reactivity presumably corresponds to different multimeric forms of the hemagglutinin and shows the high level of purity. Whether the faint band seen between 10 and 15 kDa is related to the reactivity seen with the same plasma samples after DF1-immunoprecipitation remains to be clarified.
